# Identification of glycolysis genes signature for predicting prognosis in malignant pleural mesothelioma by bioinformatics and machine learning

**DOI:** 10.3389/fendo.2022.1056152

**Published:** 2022-11-29

**Authors:** Yingqi Xiao, Wei Huang, Li Zhang, Hongwei Wang

**Affiliations:** ^1^ Department of Pulmonary and Critical Care Medicine, Dongguan Tungwah Hospital, Dongguan, Guangdong, China; ^2^ Department of Orthopaedics, Dongguan Tungwah Hospital, Dongguan, Guangdong, China

**Keywords:** malignant pleural mesothelioma (MPM), glycolysis, prognostic risk model, gene set enrichment analysis (GSEA), machine learning

## Abstract

**Background:**

Glycolysis-related genes as prognostic markers in malignant pleural mesothelioma (MPM) is still unclear. We hope to explore the relationship between glycolytic pathway genes and MPM prognosis by constructing prognostic risk models through bioinformatics and machine learning.

**Methods:**

The authors screened the dataset GSE51024 from the GEO database for Gene set enrichment analysis (GSEA), and performed differentially expressed genes (DEGs) of glycolytic pathway gene sets. Then, Cox regression analysis was used to identify prognosis-associated glycolytic genes and establish a risk model. Further, the validity of the risk model was evaluated using the dataset GSE67487 in GEO database, and finally, a specimen classification model was constructed by support vector machine (SVM) and random forest (RF) to further screen prognostic genes.

**Results:**

By DEGs, five glycolysis-related pathway gene sets (17 glycolytic genes) were identified to be highly expressed in MPM tumor tissues. Also 11 genes associated with MPM prognosis were identified in TCGA-MPM patients, and 6 (COL5A1, ALDH2, KIF20A, ADH1B, SDC1, VCAN) of them were included by Multi-factor COX analysis to construct a prognostic risk model for MPM patients, with Area under the ROC curve (AUC) was 0.830. Further, dataset GSE67487 also confirmed the validity of the risk model, with a significant difference in overall survival (OS) between the low-risk and high-risk groups (P < 0.05). The final machine learning screened the five prognostic genes with the highest risk of MPM, in order of importance, were ALDH2, KIF20A, COL5A1, ADH1B and SDC1.

**Conclusions:**

A risk model based on six glycolytic genes (ALDH2, KIF20A, COL5A1, ADH1B, SDC1, VCAN) can effectively predict the prognosis of MPM patients.

## Introduction

Malignant pleural mesothelioma (MPM) refers to a primary tumor originating from pleural mesothelial cells. The age of onset tends to be 50-70 years, and most of them are male (1). Existing studies confirmed that exposure to asbestos is the primary and definite cause of MPM. Asbestos can stimulate the body to produce induced inflammatory factors and damage genetic material; oxidative stress is involved in the formation of MPM (2). The U.S. Centers for Disease Control (CDC) identified 45,221 MPM-related deaths from, 1999 to, 2015, and the number of deaths attributed to MPM increased by 4.8% in 16 years. With the development of industry in Southeast Asia, asbestos is used more extensively in production and life, and the incidence of MPM is increasing year by year as well. MPM exhibits an insidious onset, high degree of malignancy, poor prognosis, as well as short survival. The median survival time of only supportive treatment is only 6-8 months, and the median survival time after comprehensive treatment is only 12-16 months (3). Accordingly, early diagnosis and early treatment are the main means to treat the disease, whereas there are few clinical biomarkers capable of effectively predicting the prognosis of MPM cases (4). Therefore, the related biomarkers for the prognosis of MPM should be explored.

Glycolysis refers to an important reaction stage of cellular respiration, i.e., the first step of most carbohydrate catabolism (5). Glycolysis is a special metabolic pathway that mostly occurs in the cytoplasm, so it does not require the participation of oxygen molecules. The increase in glycolysis can produce ATP for cancer cells, which has become the main source of energy for cancer cell growth and metabolism. Moreover, variations in energy metabolism are considered “hallmarks of cancer” (6). Current studies suggested that genes related to the glycolysis pathway are involved in the occurrence, invasion and metastasis of tumors and are significantly associated with the prognosis of cases (7, 8). The immortal proliferation of tumor cells causes the cell interior to be often in a state of hypoxia. The glycolysis pathway is capable of improving the tolerance of tissue cells to hypoxia and avoiding apoptosis induced by oxidative phosphorylation (9). Second, the glycolysis pathway leads to the increased lactic acid, which can also break down and destroy the cell matrix around tumor cells to promote tumor cell migration and spread to distant places (10). In addition, machine learning (ML) integrates medicine, computer science and statistics. ML can handle large, complex and disparate sources of data to assist in customizing personalized medicine and computer-aided diagnosis (11).

**Figure 1:**
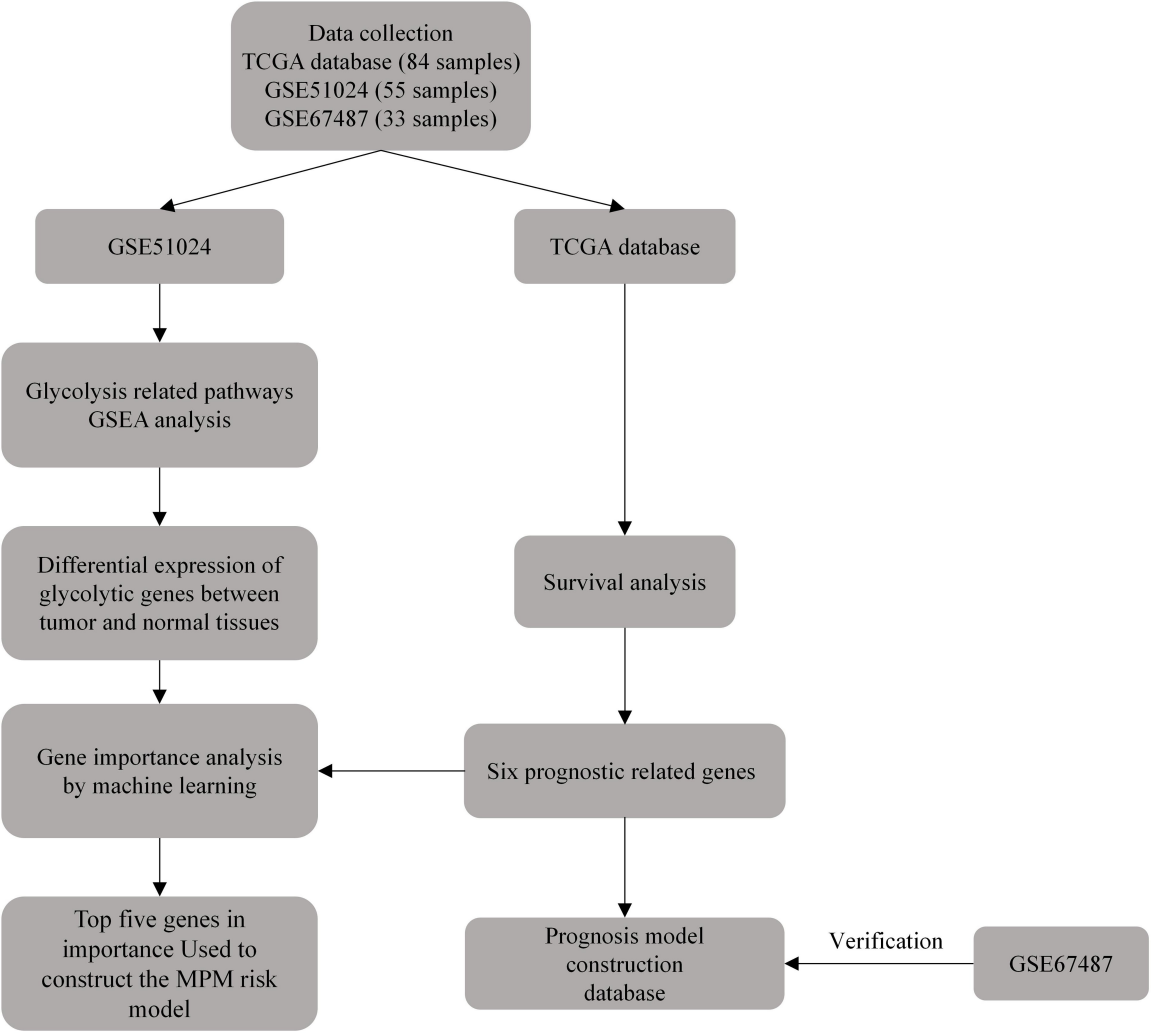
Schematic diagram of the flow of this study.

The existing prognosis of MPM still lacks effective prediction methods, and the relationship between its prognosis and glycolytic pathway-related genes remains unclear. Hopefully, this study can use bioinformatics methods and ML to study the relationship between glycolytic pathway-related genes and the prognosis of MPM cases, identify prognostic-related genes, and build a MPM prognostic risk model to provide references for patient survival assessment (**Figure 1**).

## Materials and methods

### Patient clinical dataset download and standardized analysis

The clinical information and mRNA sequencing data of MPM cases were downloaded through The Cancer Genome Atlas (TCGA) database, and 3 cases with missing survival information or sequencing data were eliminated. There was a total of 3 conditions, including 84 MPM cases. The datasets, GSE67487 and GSE51024, were obtained from Gene Expression Omnibus (GEO). **Table 1** lists the specific information of the included dataset. The mRNA data of the samples were standardized with log 2 with R 4.0.2 software limma package, and the average value of genes with multiple probes was determined.

**Table 1:** Basic characteristics of the gene expression profile data.

Dataset	Platform	Normal	MPM
TCGA	Illumina HiSeq	0	84
GSE51024	GPL570[HG-U133_Plus_2] Affymetrix Human Genome U133 Plus 2.0	41	55
GSE67487	GPL10123 Agilent-022060 SurePrint G3 Human CGH Microarray 4x1	0	33

### Gene set enrichment analysis

Through GSEA (http://software.broadinstitute.org/gsea/index.jsp), it was adopted to determine the gene set of glycolysis related pathways presented by Molecular Signatures Database (MSigDB). GSEA was performed on the dataset GSE51024 to study the expression differences of glycolysis-related pathway gene sets between tumor and normal samples. P < 0.05 was set as the critical value.

### Differentially expressed genes

312 human glycolysis-related genes were obtained through the glycolysis-related pathway gene set presented by the MSigDB database. Next, the limma package was used to identify the differentially expressed glycolytic genes between the dataset GSE51024 -MPM tissue and normal tissues. This genes with log2 fold-change (FC) > 1 and regulated P < 0.05 were considered DEGs.

### MPM prognostic gene screening and risk model construction

Next, the MPM dataset and dataset GSE51024 were extracted in the TCGA database to screen for differentially expressed genes. In addition, through the R language survival package Single-factor COX regression analysis, glycolytic genes significantly related to the overall survival (OS) of MPM cases (P < 0.05) were screened out. Through Multi-factor COX analysis, independent prognostic genes were screened, and the patient’s prognostic risk model was built simultaneously, and a nomogram was generated. *Risk Score=expmRNA1×β1+expmRNA2×β2+……+expmRNAn×βn (Exp: expression level; β is the regression coefficient of Multi-factor COX analysis)*.

### Assessment and verification of predictive significance of MPM prognostic risk model

Lastly, the risk score of MPM cases was determined by using the built prognostic risk model. Cases fell to high-risk and low-risk groups based on the median value. R software survival and survminer packages were adopted to draw Kaplan-Meier (K-M) curve and ROC curve to assess the predictive significance of the prognostic model. For the dataset GSE67487, K-M curve and ROC curve were also plotted by complying with the prognostic model.

### Further screening of prognostic genes by machine learning

Next, six independent prognostic risk genes were further screened. A specimen classification model was constructed using support vector machine (SVM) and random forest (RF) to predict the risk of MPM. Briefly, first, a clustering analysis is performed based on the differential expression values of six prognostic genes in normal and tumor tissues based on the GSE51024 dataset. Then, the performance of different types of samples is evaluated by iterating the combination of random features until the optimal combination of features is obtained for constructing the risk model. The RF model was additionally used to determine the feature importance (FE) of the variables (FE was assessed based on the out-of-bag error rate, reflecting the contribution rank of each gene when classifying MPM tumor tissue versus normal control tissue).

### Statistical analysis

All data were analyzed using R 4.0.2 (http://www.R-project.org). Single-factor COX regression and Multi-factor COX analyses were used to analyze the prognostic risk of glycolytic genes and tumor patients, and survival differences between high- and low-risk groups were analyzed by log-ranking tests defined by K-M analysis. ROC curves were used to test the diagnosticity of risk models. P < 0.05 was considered a significant difference.

## Results

### Glycolysis functional pathway acquisition and differential gene screening

**Figure 2:**
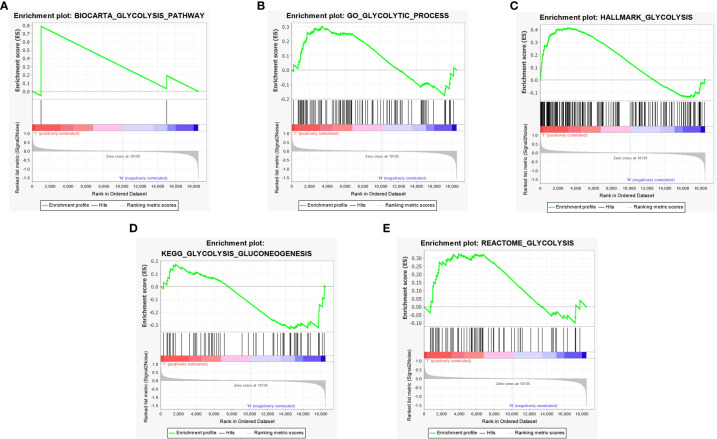
GSEA identified that five glycolysis gene sets were significantly enriched. **(A)** BIOCARTA GLYCOLYSIS. **(B)** GO GLYCOLYTIC PROCES. **(C)** HALLMARK GLYCOLYSIS. **(D)** KEGG GLYCOLYSIS GLUCONEOGENESIS. **(E)** REACTOME GLYCOLYSIS.

**Figure 3:**
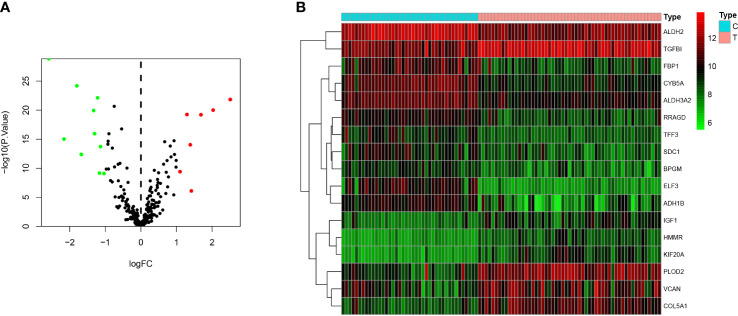
Differentially expressed genes between MPM and normal tissues. **(A)** The volcano plot of 17 differentially expressed genes (The red dots represent the level of high expression and the green dots represent the level of low expression). **(B)** Heatmap of 17 differently expressed genes (The depth of red represents the level of high expression, and the depth of green represents the level of low expression).

A total of 5 glycolysis-related pathway gene sets were obtained from the MSigDB, including BIOCARTA GLYCOLYSIS PATHWAY, GO GLYCOLYTIC PROCES, HALLMARK GLYCOLYSIS, KEGG GLYCOLYSIS GLUCONEOGENESIS, REACTOME GLYCOLYSIS. Next, GSEA was performed on the dataset GSE51024, and it was found that the five glycolysis-related pathway gene sets were significantly different in MPM tissue and normal samples, and were positively correlated with MPM tissue (P < 0.05; **Figures 2A–E**). There are a total of 312 genes in the 5 glycolysis-related pathway gene sets, and 17 glycolysis genes that are differentially expressed between the dataset GSE51024-MPM tissue and normal tissues were screened out using the limma package (P < 0.05; **Figures 3A, B**).

### Prognostic gene screening and risk model construction of glycolysis for MPM

**Figure 4:**
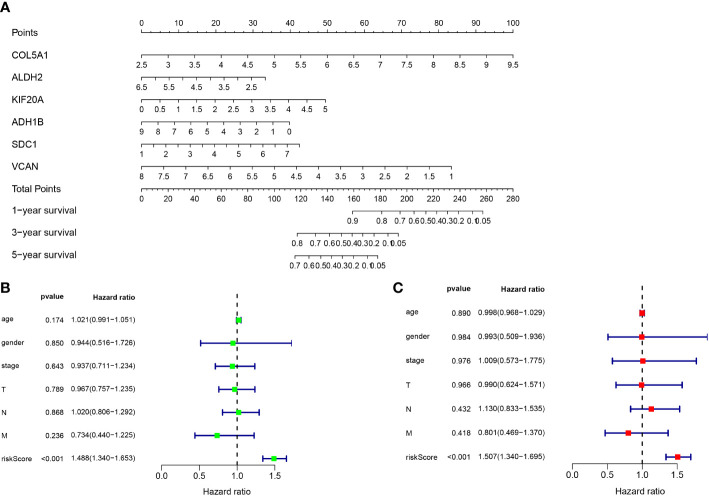
**(A)** Nomogram of prognostic model. **(B)** Single-factor COX regression analysis. **(C)** Multi-factor COX analysis.

The gene sequencing data of TCGA mesothelioma cases were sorted through R language and extracted to obtain the 17 differential gene expression profiles of the dataset GSE51024. Moreover, through Single-factor COX regression analysis, 11 glycolytic pathway-related genes were found to be significantly associated with the overall survival (OS) of the patient (P < 0.05). Lastly, through Multi-factor COX analysis, 6 genes (COL5A1, ALDH2, KIF20A, ADH1B, SDC1 and VCAN) were lastly included to build a patient prognostic risk model, and a nomogram was drawn simultaneously (**Figure 4A**), To be specific, COL5A1, ALDH2, KIF20A, ADH1B, SDC1 and VCAN are independent risk genes (**Table 2**). Furthermore, a Single-factor COX regression analysis and a Multi-factor COX analysis combined with TCGA clinical information identified the risk score as an independent prognostic risk factor (P<0.05, **Figures 4A, B**). *Riskscore = (COL5A1×0.487)*+*(ALDH2×-0.252)+(KIF20A×0.337)+(ADH1B×-0.151)+(SDC1×0.223)+(VCAN×-0.406)* (**Figures 4B, C**).

**Table 2:** Characteristics of genes in the prognostic model.

Gene	Univariate analysis			Multivariate analysis			Coefficients
	HR	95%CI	P-value	HR	95%CI	P-value	
COL5A1	1.483	1.267-1.743	<0.001	1.627	1.107-2.393	0.013	0.487
ALDH2	0.506	0.383-0.670	<0.001	0.777	0.559-1.081	0.134	-0.252
KIF20A	1.811	1.442-2.276	<0.001	1.401	1.092-1.798	0.008	0.337
ADH1B	0.743	0.660-0.836	<0.001	0.860	0.740-0.999	0.049	-0.151
SDC1	1.458	1.214-1.750	<0.001	1.249	1.009-1.547	0.041	0.223
VCAN	1.290	1.107-1.502	0.001	0.666	0.462-0.961	0.030	-0.406

### Assessment and verification of predictive significance of MPM risk model

**Figure 5:**
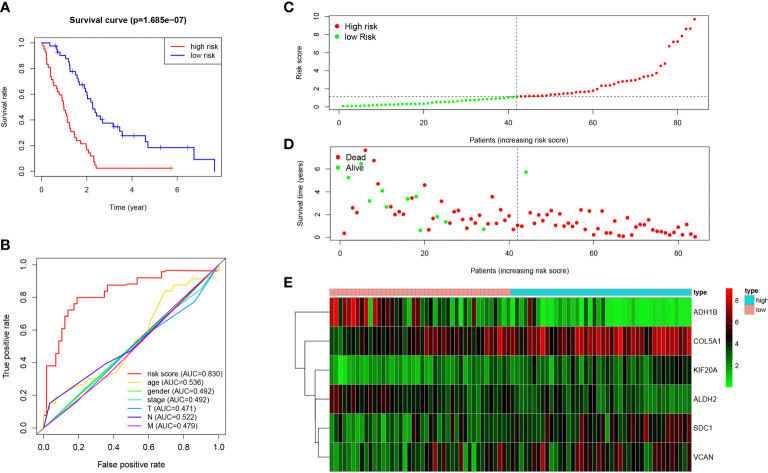
Prognosis of high-risk and low-risk MPM cases. **(A)** K-M analysis of MPM cases is stratified by median risk. High risk scores are associated with general poor survival. **(B)** Multi-index ROC curve of risk score and other indicators. **(C)** Risk score distribution of low-risk (green) and high-risk (red) in MPM cases. **(D)** Scatter plot of survival status of MPM cases in. Red dots (dead); green dots (alive). **(E)** Expression of risk genes in the high-risk (blue) and low-risk (pink) of the OS model.

The risk score of each patient in the TCGA dataset was calculated through the built MPM risk model, and the cases fell to high and low risk groups based on the median risk value. The K-M curve showed that the survival rate of the high-risk group was significantly lower than that of the low-risk group (P < 0.05; **Figure 5A**). The ROC curve shows that Area under curve (AUC)=0.830, which has a significant prognostic significance relative to age, gender, and tumor stage (P < 0.05; **Figure 5B**).

**Figure 6:**
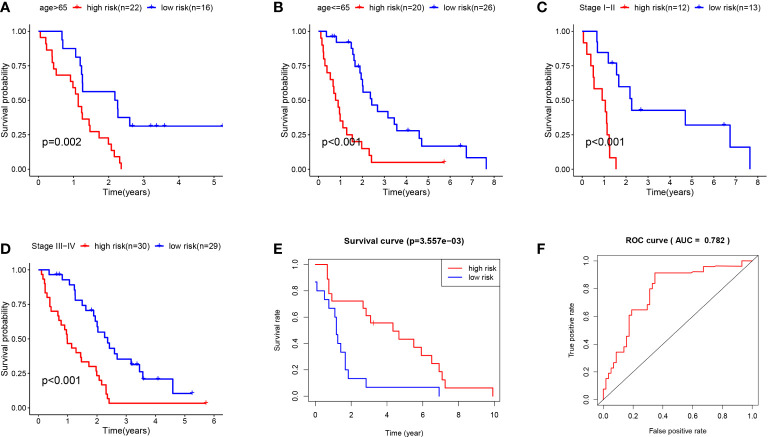
**(A)** K-M curve of TCGA MPM patients younger than 65. **(B)** KM curve of TCGA MPM patients older than 65. **(C)** K-M curve of TCGA MPM patients with stage I-II. **(D)** KM curve of TCGA MPM patients with stage III-IV. **(E)** K-M curve of GSE67487 patients. **(F)** ROC curve analysis of GSE67487 patients.

Besides, the survival rate distribution was analyzed by ranking the risk scores of all MPM cases (**Figure 5C**). From the scatter plot, we find that with the increase in the risk score, the patient’s mortality rate gradually rises (**Figure 5D**). Genes with HR > 1 (COL5A1, KIF20A, SDC1) was defined as dangerous genes, and genes with HR < 1 (ALDH2, ADH1B, VCAN) as protective genes. Cases in high-risk populations are more likely to express risk genes, and those in low-risk populations are inclined to express protective genes (**Figure 5E**). Furthermore, as suggested by conducting the clinical subgroup analysis, for different age stratifications and tumor stages, the survival rate of the high-risk group based on the prognostic model of the K-M curve was also significantly lower than that of the low-risk group (P < 0.05; **Figures 6A–D**). In the dataset GSE67487 K-M curve, the survival rate of the low-risk group was significantly higher than that of the high-risk group, ROC curve AUC = 0.782, which verified the reliability of the prognostic model (P < 0.05; **Figures 6E, F**).

### Further screening of MPM prognostic genes by SVM and RF

**Figure 7:**
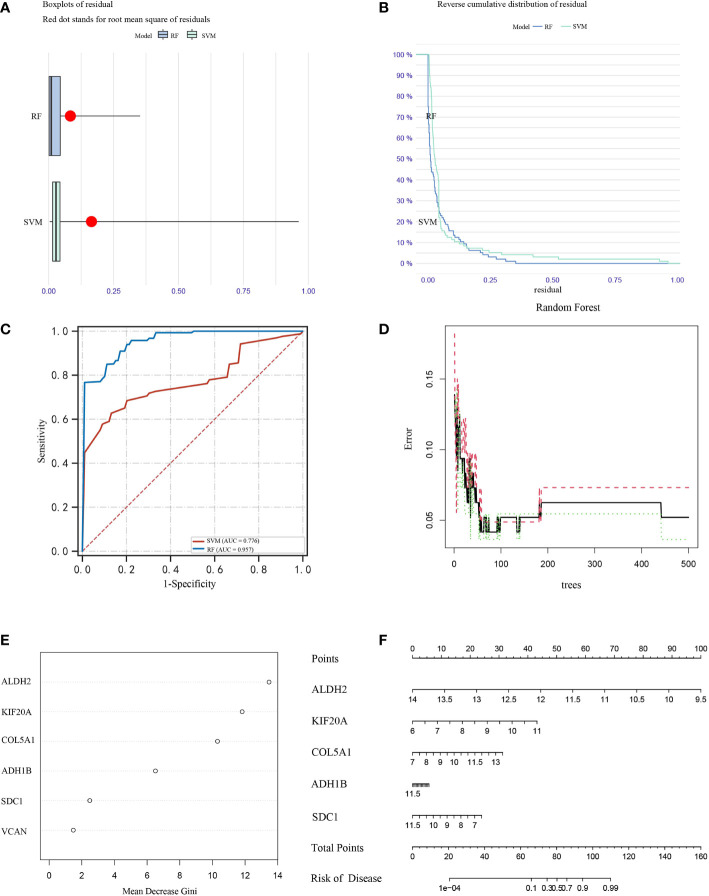
Box plots **(A)** and error analysis **(B)** of two unsupervised clustering methods for MPM-based differential expression of prognostic genes, and comparison of the accuracy **(C)** of the two classification modes, with the RF classification mode **(D)** classifier iteration process. **(E)** Ranking of the importance of prognostic genes in correlation with the risk of MPM prevalence, **(F)** disease models were constructed for the five prognostic genes with the highest correlation with the risk of MPM prevalence.

COL5A1, ALDH2, KIF20A, ADH1B, SDC1 and VCAN genes were selected for inclusion in the analysis, and SVM and RF classification models were constructed based on the optimal feature gene combinations (**Figures 7A, B**). After analysis, the results showed that the best prognostic gene combination had the highest classification transfer accuracy when the number of prognosis was set to 5. In addition, the RF classification model had higher accuracy compared to the SVM (AUC=0.957 *vs.* AUC=0.776; P < 0.05; **Figure 7C**). The iterative calculation process of the RF classification model is shown in **Figure 7D**. The RF classification model algorithm obtained the specific importance ranking of prognostic genes in terms of MPM prevalence correlation (**Figure 7E**), and finally screened to obtain the five prognostic genes with the highest correlation with MPM prevalence risk The five prognostic genes with the highest risk of MPM (ALDH2, KIF20A, COL5A1, ADH1B and SDC1 in order of importance) were finally screened, and the MPM risk model was constructed based on the above five genes (**Figure 7F**).

## Discussions

Over the past few years, some researchers have confirmed that age, gender, smoking history, tumor size, pathological stage, lymph node metastasis and distant organ metastasis and other clinicopathological features are of critical significance for the prognosis of cancer cases. However, the prognosis of tumors at the genetic level exhibits higher accuracy, and it facilitates targeted and immunotherapy and can help clinicians choose the optimal treatment strategy (6). MPM refers to an aggressive disease with unique morphology and distribution. Due to its special growth pattern, clinical staging is difficult. Traditionally, age, sex, contact, tumor size, radiological evidence, pathological staging and others face difficulty in achieving the accurate prognosis of cases (12). As confirmed by existing studies, glycolysis displays a close relationship to the occurrence, migration and metabolism of malignant tumors, and genes related to glycolysis are inseparable from the regulation of tumor metabolism, proliferation and differentiation (13). MPM cells commonly show higher rates of glucose uptake and glycolysis, while the amount of lactic acid infiltrating into the gap is elevated, and the entry and exit of lactic acid into and out of the cell is critical to maintain intracellular PH stability and glycolysis. Earlier studies have confirmed that the expression of monocarboxylate transporters (MCTs) and the chaperone basigin (CD147). Lactate in and out of cells plays a vital role of assessing the progress of MPM and can act as a molecular marker for disease prognosis (14).

In the present study, we lastly identified 6 glycolysis-related genes (COL5A1, ALDH2, KIF20A, ADH1B, SDC1, VCAN), and verified the prognostic significance for the mentioned 6 genes for MPM cases through Single-factor COX regression analysis and Multi-factor COX analysis. The K-M analysis also shows that high-risk scores are related to metastasis and poor prognosis.

The COL5A1 gene is capable of encoding a low-abundance fibrous collagen α chain. Collagen fiber molecules are trimers and can be composed of one or more α chains. COL5A1 is a member of the collagen family, and collagen is the most abundant component in the extracellular matrix (ECM). They provide structural integrity and tensile strength for human tissues and organs (15). In cancer development, collagen constantly affects the physical and biochemical characteristics of the tumor microenvironment, as well as regulating the polarity, migration and signal of cancer cells (16). COL5A1 encodes the α chain of type V collagen, which exists in tissues containing type V collagen and regulates the assembly of heterotypic fibers composed of type I and type V collagen. Cheon et al. found that COL5A1 is regulated by TGF-β1 signaling. This up-regulation of COL5A1 can promote the metastasis and overall survival rate of cases with serous ovarian cancer (17). Shengjun S et al. also identified COL5A1 as a marker for poor prognosis of bladder cancer through Weighted Gene Co-expression Network Analysis (WGCNA) (18). Moreover, existing studies confirmed COL5A1 as a potential core gene to promote metastatic renal cell carcinoma (19). The present study reported that the COL5A1 gene in MPM tissues was significantly up-regulated, undoubtedly demonstrating that COL5A1 can promote the transfer of MPM.

Aldehyde dehydrogenase 2 (ALDH2) refers to a vital mitochondrial enzyme controlling ethanol metabolism. ALDH2 gene polymorphism displays a close relationship to the susceptibility of colorectal cancer, esophageal cancer, liver cancer and other cancers. In particular, the mutation of ALDH2 gene is closely associated with the risk of cancer. As a novel biomarker, ALDH2 has suggested a very attractive prospect in the screening, diagnosis and prognosis assessment of various diseases (20). ALDH2 is a 56 kDa tetrameric protein and highly polymorphic enzyme with the same subunits. Each of the four polymer subunits contains the structure of three main domains: the catalytic domain, the coenzyme or NAD+ binding domain, and the oligomerization domain (21, 22). ALDH2, a vital oxidative stress molecule, is capable of reducing the production of reactive oxygen species (ROS), thereby preventing cell apoptosis and cell damage attributed to hyperoxia or acetaldehyde (23). Specific to the esophagus, gastrointestinal tumors and liver cancer closely related to drinking display a tight association (24, 25). As suggested by Park et al., smokers with ALDH2 genotype are subject to a higher risk of lung cancer. However, no independent risk factor is identified between lung cancer and ALDH2 polymorphism. There is more research to be done on this issue (26). Clinically, ALDH2 has great prospects in tumor diagnosis and can initially detect the human ALDH2 genotype; given whether the patient’s genes are susceptible to cancer, cases are given some reasonable treatment suggestions to achieve individual precision medicine (27). Likewise, alcohol dehydrogenase (ADH) is also critical to ethanol metabolism. ADH is a dehydrogenase superfamily located on chromosome 4q22-q24, covering class I (ADH1A, ADH1B and ADH1C) and class II (ADH4), Class III (ADH5), Class IV (ADH6) and Class V (ADH7) (28). Existing studies have reported that members of the ADH gene family are closely related to the prognosis of various cancers (29), and genetic mutations in ADH affect the risk of cancer in alcohol-dependent individuals as well (30). According to Liu et al., the expression levels of ADH1A, ADH1B, ADH1C, and ADH6 decreased significantly with the aggravation of liver cancer (31). In addition, existing studies indicated that ADH1B has a good prognostic significance for pancreatic cancer as well (32). Existing studies have shown that the expression levels of ALDH, ADH1B and the risk of poor prognosis of cancer were negatively correlated, and the high level of ALDH, ADH1B expression also implied a higher survival rate of MPM patients.

Kinesin Family Member 20A (KIF20A) is considered one of the vital factors of mitosis. As revealed from numerous recently conducted studies, KIF20A is considered a vital gene for considerable tumors (e.g., hepatocellular carcinoma or ovarian cancer) (33, 34). The relationship between KIF20A and MPM is also very close. Xiangxin Z et al. proved through bioinformatics that the survival rate of MPM cases in the KIF20A high expression group was significantly lower than that of the low expression group. In addition, as indicated by the analysis of Cox regression factors, as opposed to MPM cases in the low expression group, the high expression of the mentioned genes is a risk factor for prognosis (35). Furthermore, the present study proved that the survival time of MPM cases with high KIF20A expression was significantly shorter than that of the low expression group, complying with the results of this article.

Syndecan-1 (SDC-1) refers to a proteoglycan, critically impacting the occurrence and development of MPM *via* its heparan sulfate (HS) chain as a co-receptor (36). It is capable of combining with basic fibroblast growth factor (bFGF) to regulate the formation of new blood vessels. MPM is recognized as one of the most aggressive tumors known, expressing high levels of angiogenic growth factors. As suggested from the existing studies, the high expression of SDC-1 can significantly promote the microvessel density in MPM tumors and promote tumor migration (37). Szatmári T et al. found that in MPM, the expression of SDC-1 is related to epithelioid morphology and the inhibition of growth and migration. Moreover, the overexpression of SDC-1 is involved in the regulation of cell growth, cell cycle progression, adhesion, migration and extracellular matrix. The genes of the tissue have a profound impact, which is an important prognostic indicator of MPM (38). Versican (VCAN) refers to a vital protein in the ECM, capable of accumulating in the tumor stroma; it can significantly regulate the malignant transformation of tumors and the progression of tumors as well (39). Moreover, VCAN has been confirmed to display a close relationship to the survival, development and recurrence of numerous malignant tumors. For instance, VCAN is capable of promoting the migration of breast, gastric and prostate cancer, and its expression level can determine the prognosis of malignant tumors (40). Interestingly, our study found that high expression of VCAN implies better prognostic survival of MPM. Therefore, how VCAN specifically regulates the physiological activities of tumor cells remains to be further explored.

Compared to traditional medical statistics methods, ML typically has higher efficacy for disease diagnosis than traditional methods, is more widely applicable, and can rank the importance of impact, which provides a statistical basis for screening the core variables that have the greatest impact on outcomes. In this study, we also ranked the prognostic importance of six bioinformatically screened glycolytic genes by ML, and finally identified five genes that mainly affect the prognosis of MPM, in descending order of importance: ALDH2, KIF20A, COL5A1, ADH1B and SDC1. Of course, there is a need for more advanced learning methods such as Neural networks, Deep learning and Decision tree learning to further develop accurate prognostic models for diseases, which are all important directions for the future of artificial intelligence in medicine.

The present study has several limitations. First, the databases involved in this study, including TCGA, MSigDB and GEO, among others, were mainly included in the North American population, and the validity of this prediction model outside North America needs further validation. Second, these identified glycolytic genes could serve as prognostic biomarkers and novel therapeutic targets for MPM, but further *in vitro* functional analysis of MPM cell lines is still needed to better understand the role of these putative genes. On the whole, risk-of-use models constructed based on glycolytic genes are suitable as reference information for clinicians and do not represent an absolutely accurate prognosis. In the future, more effective and convenient tools should be developed to help clinicians analyze the risk of MPM prognosis.

## Conclusions

In brief, the present study built a novel prognostic model of six glycolysis-related genes (i.e., COL5A1, ALDH2, KIF20A, ADH1B, SDC1 and VCAN) for the prognosis of MPM cases, which is an important reference for treating MPM cases and developing targeted drugs.

## Data availability statement

The original contributions presented in the study are included in the article/supplementary material. Further inquiries can be directed to the corresponding author.

## Author contributions

YQX carried out the acquisition and interpretation of data and was the major contributor to drafting the manuscript. WH & YQX were responsible for data statistics and analysis. LZ & HWW were responsible for guiding the clinical knowledge of MPM. WH contributed to the article’s ideas and reviewed the manuscript. All authors contributed to the article and approved the submitted version.

## Conflict of interest

The authors declare that the research was conducted in the absence of any commercial or financial relationships that could be construed as a potential conflict of interest.

## Publisher’s note

All claims expressed in this article are solely those of the authors and do not necessarily represent those of their affiliated organizations, or those of the publisher, the editors and the reviewers. Any product that may be evaluated in this article, or claim that may be made by its manufacturer, is not guaranteed or endorsed by the publisher.
